# Effects of an online mindfulness-based program for parents of children with attention deficit/hyperactivity disorder: a pilot, mixed methods study

**DOI:** 10.3389/fpsyt.2024.1376867

**Published:** 2024-04-24

**Authors:** Herman Hay Ming Lo, Jason Lam, Zoe Ji-wen Zhang, Marsena Cheung, Stanley Kam Chung Chan, Elisabeth Wai Yin Wong, Susan M. Bögels, Wing Hong Chui

**Affiliations:** ^1^ Hong Kong Polytechnic University, Hong Kong, Hong Kong SAR, China; ^2^ Professional Practice and Assessment Centre, Hong Kong Polytechnic University, Hong Kong, Hong Kong SAR, China; ^3^ University of Southampton, Southampton, United Kingdom; ^4^ The Chinese University of Hong Kong, Hong Kong, Hong Kong SAR, China; ^5^ Hong Kong College of Psychiatrists, Hong Kong, Hong Kong SAR, China; ^6^ UvA minds Academic Treatment Centre for Parent and Child, University of Amsterdam, Amsterdam, Netherlands

**Keywords:** mindfulness-based intervention, online, attention deficit/hyperactivity disorder, parents, mixed methods study

## Abstract

**Objectives:**

Online mindfulness-based program (MBP) for parents and families especially in clinical population is limited. Engagement and significant dropout are major issues in MBP implementation. This pilot study examined the effects of an online mindfulness-based program (MBP) on parents of children with Attention Deficit/Hyperactivity Disorder (ADHD).

**Methods:**

A mixed methods study was applied to evaluate the effects of the MBP. A total of 43 parents were recruited and were randomly assigned into intervention group and waitlist control group. The online MBP lasted for 28 days, including 20 psychoeducation videos, homework audio guidance, and four instructor-led online group meetings. Purposive sampling was used to recruit parents who completed the program to share their experiences and suggestions for improving the program in semi-structured online interviews.

**Results:**

Quantitative data showed that participants from the online MBP reported a medium to large effect on the reduction of child ADHD symptoms. In semi-structured interviews, participants reported positive experiences in their help seeking intention, and personal changes, such as emotion regulation and quality attention to their children. Participants further made suggestions for improvement.

**Conclusions:**

The effect of online MBP is promising, and the program should be conducted. A large scale randomized controlled trial should be conducted to investigate the effects of MBP in clinical populations.

**Clinical trial registration:**

ClinicalTrials.gov NCT05480423.

## Introduction

Attention Deficit/Hyperactivity Disorder (ADHD) is one of the most common developmental disorders, with a prevalence of up to 5% (1). A national survey in 2016 (n = 45,736) estimated that 9.4% children aged 2 -17 years of age in the United States had ever received an ADHD diagnosis (2). In addition, ADHD is a disorder that persists and is present even in adulthood (3). The primary symptoms of ADHD include inattention, hyperactivity, and impulsivity, which limit social and academic functioning. Most children with ADHD have significant impairments in executive functions (EFs), which include cognitive flexibility, inhibition, self-control, self-regulation, working memory, problem-solving, reasoning, and planning (4). In particular, when some children with ADHD exhibit hyperactive and impulsive behaviors, they are more prone to engaging in physical and relational aggression, as well as facing a higher likelihood of peer rejection (5, 6), compared to children without ADHD.

Childhood ADHD has been associated with poor family functioning, increased stress within the family, higher rates of parental psychopathology, and increased conflicts in parent–child relationships (7). Challenging child behavior evokes harsh parenting responses, including intense hostility and negative emotionality, which are often exacerbated by children’s comorbid oppositional and conduct problems in a mutually reinforcing process (8). Parents of children with ADHD have reported higher levels of parenting stress, lower levels of social support and quality of life, and greater marital conflicts than parents of children without ADHD (9, 10). Considering the reciprocal dysfunctional interactions between children with ADHD and their parents, interventions should focus on improving parents’ understanding of ADHD symptoms in children. Additionally, these programs should aim to enhance parents’ knowledge and competence in managing their child’s behaviors and promoting overall functioning of all family members (11).

### Treatments for children with ADHD

Pharmacotherapy is the first-line treatment for children with ADHD. Most children with ADHD experience improvements after treatment with psychostimulant medication, particularly in behavior compliance and attention maintenance (12). However, some children demonstrate side effects such as poor appetite, sleeping problems, and negative mood (13). Behavioral interventions, including antecedent-based strategies, contingency management techniques, and self-management skills, are commonly used to manage the disruptive behaviors of children with ADHD (14). However, parents of children with ADHD with high levels of stress are more likely to encounter challenges and frustrations in response to children’s reactions, resulting in sequential rejection, negative emotionality, and a dysfunction of their parenting roles. This, in turn, may reduce their capacity to effectively apply the techniques taught in parent behavioral training programs (15). While behavioral training may confer short-term benefits, its long-term effects are still uncertain, as children with ADHD cannot learn self-regulation without parental supervision (16). More options for psychological interventions that would target the well-being of the entire family system are recommended.

### Application of a mindfulness-based programs in parents and families

Mindfulness is a process of regulating attention to bring a quality of non-elaborative awareness to current experience and a quality of relating to one’s experience within an orientation of curiosity, experiential openness, and acceptance (17). Over the last four decades, mindfulness-based programs (MBPs) have been applied to improve the mental health of individuals with chronic medical conditions, based on their ability to strengthen individuals’ coping strategies (18, 19). In MBPs, individuals practice mindfulness exercises including body scan, mindful stretching, mindful sitting, and other informal exercises with the guidance of mindfulness-based instructors that can help them to enhance their coping abilities in daily life.

A recent systematic review reported that MBPs showed a small to moderate reduction in parenting stress at the post-intervention and 2-month follow-ups (20). Another review reported that a child–parent parallel group MBPs conferred limited positive benefits to family functioning, parental mental health and child mental health (21). Both reviews showed a growing interest in applying MBPs in child and family contexts. Recently, two randomized controlled trials (RCTs) were conducted specifically for children with ADHD and their parents. Siebelink et al. (22) compared an 8-week family MBP (*n* = 55) with care-as-usual (*n* = 48). In this study, the family MBP conferred small but statistically non-significant post-treatment improvements in child self-control and ADHD symptoms. Moreover, parents reported significant improvements in mindful parenting. With a similar program intensity, Lo et al. (23) compared a family MBP (*n* = 50) with a waitlist control group (*n* = 50) based on a sample of Chinese families. Families after MBP reported significant greater improvements in children’s ADHD symptoms and parenting stress than those from waitlist control group. The above findings suggest that MBPs is a promising approach to supplement pharmacotherapy in treating children with ADHD and their parents.

### Applying technology in an MBP to support parents of children with ADHD

Practitioners and researchers have collaborated to apply technology and convert face-to-face MBPs into online or app-based MBPs during and after the impact of COVID-19. Studies have either recruited healthy parents (24, 25) or conducted open trials (26, 27). In view of the above literature, we identify a few limitations in the online mindfulness-based treatment for children and families. First, among the existing studies, most have had a weak study design, marked by a small sample size and a lack of control group. As such, the results of these preliminary studies cannot be used to assess MBP effectiveness. Second, most intervention targeted healthy parents and their effects on clinical populations such as children with ADHD and their parents is uncertain. Third, engagement is the key to success for online treatment, and parents of children with ADHD face multiple demands and challenges related to work–family balance, complicating their commitment to MBPs and following mindfulness exercises on a regular basis. One study of online MBP for parents reported a low completion rate below 30% (26). Adaptations should be made for improving service utilization based on the nature of the target populations (28). Attempts at providing daily digital prompts (29), mentor phone support (30), and guidance on demand (31) have been less costly, but the impacts on engagement have been limited and non-significant. Reviews have emphasized the improved outcomes associated with professional support when implementing technological-based interventions. This is particularly relevant for clarifying expectations regarding the role and availability of professionals throughout the program, managing emergencies between sessions, and ensuring sufficient audio and camera quality to establish rapport (32). Many parents of children with ADHD have also shown deficits in attention (22, 33). Therefore, such new online interventions should include multiple components integrating technology intervention with low intensity professional support to enhance the motivation to complete mindfulness exercises.

Our research team modified the ordinary MBP structure which normally included weekly meeting lasting 2 hour or longer, while the new program structure integrates short daily online psychoeducation videos with audio mindfulness guidance each lasting 3 to 15 minutes only. The first feasibility study was a self-help program that involved healthy parents and they were randomly assigned to intervention group (*n* = 136) and waitlist control group (*n* = 137). Findings suggested that the program was efficacious in improving parents’ mindfulness and well-being and in reducing their anxiety symptoms (34). In this study, we further modified the program to address the needs of parents and children with ADHD. This study investigated the effects of an online MBP on the outcomes of children with ADHD and their parents. The following two hypotheses were tested:

First, according to the role of MBP in attention regulation and previous reviews (20–23), we hypothesized that parents who completed the online MBP would report more significant improvement in mental health compared to parents of children with ADHD in the wait-list control group. These improvements would include reduced levels of parenting stress, depression, and anxiety, as well as increased psychological well-being.

Second, through participating in the MBP, we anticipate that parents would not only experience personal benefits but also observe a positive impact on their children. We hypothesized that children whose parents completed the online MBP, when compared to the children with ADHD in the waitlist control group would report a significant reduction in ADHD symptomology.

## Method

### Study design

Registered with ClinicalTrials.gov (NCT05480423), the pilot study employed a mixed-method design with an explanatory sequential approach to address research questions from multi-level perspectives (35). Administrating validated scales for primary outcomes measurements and conducting follow-up semi-structured interviews for subjective in-depth experience in the program provided a more comprehensive understanding of its quality, effectiveness, limitations, and areas of improvement (36).

For quantitative data, a stratified and waitlist RCT design was applied. The intervention program began in August 2022 and lasted for 28 days. The intended sample included forty parents whose children were diagnosed with ADHD. Eligible participants were randomly assigned to the online MBP or the control group. Because of the small sample size, only the stratum of presence (vs. absence) of other ADHD comorbidities was included. Those in the control group were put on a waitlist and received the same intervention after the intervention group completed the program three months later. All participants completed a questionnaire at baseline (T0), one-month follow-up (T1), and three-months follow-up (T2). The questionnaire took 20 minutes to complete.

For qualitative study, a purposeful sampling was applied. Semi-structured interviews were organized for parents who completed the program in the intervention group. They were invited to share their experiences in the online program one week after the last session in an one-hour interview via Zoom. An interview protocol is provided in **Appendix 3**. An independent MBP instructor who was not involved in the program implementation conducted the interviews avoiding reporting bias.

### Participants

Inclusion criteria included parents 1) whose children are between 6 and 18 years old and diagnosed with ADHD based on their parents’ self-report, 2) can understand and speak Cantonese Chinese, and 3) should be the primary caretaker of the children in the last one year. Exclusion criteria included parents 1) who were diagnosed with psychosis, developmental disabilities, or cognitive impairment, which may present difficulties in comprehending the content of the program, and 2) have had prior experience in any 8-week Mindfulness-Based Stress Reduction/Mindfulness-Based Cognitive Therapy (MBSR/MBCT) or equivalent program.

Initially 47 parents enrolled and were randomly assigned. One provided invalid contact and three expressed discontinuations before the data collection and the commencement of the MBP. The final sample was 43 parents. **Table 1** summarized the demographic characteristics and the differences in them between intervention and control groups. We treated the existence of other comorbidities in ADHD children as a stratum to be randomized. Despite randomization, the educational level was significantly higher in the intervention group. It was thus included in subsequent analyses as a covariate. Parents who completed the program in the intervention group were invited, and nine of them agreed to participate in the qualitative interviews.

**Table 1:** Participants characteristics.

Variables	*M* (*SD*)	Intervention		*M* (*SD*)	Control		χ^2^	*t*	*P*
		n	%		n	%			
Role							0.527		.468
Mother		22	51.2		18	41.9			
Father		1	2.3		2	4.7			
Marital status							0.380		.538
Married		20	46.5		16	37.2			
Divorced or separated		3	7.0		4	9.3			
Other ADHD comorbidities							0.020		.887
No		12	27.9		10	23.3			
Yes		11	25.6		10	23.3			
Educational level	3.78 (1.00)			3.00 (1.38)			3.628	-2.154	.037
Junior school or below		0	0.0		3	7.0			
High school		4	9.3		6	14.0			
Post-secondary (non-degree)		2	4.7		2	4.7			
Post-secondary (degree)		12	27.9		6	14.0			
Graduate school		5	11.6		3	7.0			
Household income	6.39 (3.37)			6.70 (2.87)			0.030	.321	.750
Below $10k		3	7.0		2	4.7			
$10k−$14k		1	2.3		1	2.3			
$15k−$19k		1	2.3		0	0.0			
$20k−$24k		3	7.0		1	2.3			
$25k−$29k		1	2.3		1	2.3			
$30k−$39k		2	4.7		2	4.7			
$40k−$49k		3	7.0		5	11.6			
$50k−$59k		0	0.0		2	4.7			
$60k−$79k		1	2.3		2	4.7			
$80k or above		8	18.6		4	9.3			
Age of parents	42.65(5.26)			41.80(5.39)				-.524	.603
Age of children with ADHD	10.80(3.33)			9.53(2.98)				-1.212	,234
Number of family members	2.91 (.85)			2.65 (.75)				-1.073	.290
Number of ADHD children	1.17 (.39)			1.20 (.41)				.214	.831

### Recruitment and randomization

The project was introduced to two NGOs who are specialized in working with parents of children with ADHD. Staff of the collaborating organizations distributed the program leaflets to potential participants by displaying posters and leaflets at their websites and newsletter and through their contact list. Interested parents completed a simple screening survey on Qualtrics with questions relevant to the inclusion and exclusion criteria. We prohibited Qualtrics to record multiple responses with the same IP address to prevent ineligible parents filling the survey again but with different answers. Eligible parents were placed on a contact list for coordination. Before the program began (T0), parents who confirmed their participation gave informed consent to complete a questionnaire measuring the primary outcomes. Eligible parents underwent a stratified randomization and assigned to either the intervention or the waitlist-control group using the R package *blockrand*. The package generated a list of unique IDs for each stratum (i.e., ADHD only or with comorbidities) and randomly assigned them into the minimum available block size of 2 or 4 to yield balanced sample sizes between groups. Afterwards, IDs within the same block were randomly assigned to either the intervention or control group and to participants in the respective stratum. Parents were informed and given HKD $100 (USD $12.80) voucher after completing questionnaire at T1 and T2, respectively.

### Intervention

The online program lasted for four weeks, a total of 28 days. Each week had a designated module, including *Mindfulness and focused attention*, *Mindfulness and Body*, *Mindfulness in Challenging Moments*, and *Self-care under Stress*. This study produced 20 psychoeducation videos, each lasting 5 to 10 minutes for each weekday, with links to be sent to participants. Detailed program content was summarized in **Appendix 1**. The video included mini-lecture and further instructions of mp3 files of guided mindfulness exercises. The videos were presented by three experienced MBSR/MBCT instructors. The total time spent in each session was around 15 to 20 minutes. Parents were free to consume the materials at any time they were convenient, and reviewed the materials again or finished the incomplete ones when they fell behind during the weekends. Within the 20 videos, five of them were adapted to provide guidance and demonstrate for parents to practice mindfulness with children at home.

In view of the attrition rate of previous online intervention literature, this study included four weekly one-hour online group sessions with mindfulness-based instructors. The involvement in an online group could facilitate parents to understand the shared experiences in taking care of children with ADHD such as their universal suffering, which might offer chances to improve the engagement of parents in the online treatment. Three online groups were organized at different time slots and each instructor moderated one group. During the meetings, the instructors led one of the mindfulness exercises that was introduced in psychoeducation videos, reviewed the program content, and facilitated inquiries of parent’s personal experiences and questions during practice. The waitlist-control group received the identical program three months after the intervention group began.

### Outcomes

#### ADHD symptomology

The Strengths and Weaknesses of ADHD Symptoms and Normal Behaviors (SWAN) Rating Scale was used to measure the similarity or differences between a child’s attention skills and those of the general population (37). A total of 18 items represents a range of behavioral characteristics. Half of them cover the domain of inattention (e.g., ignore extraneous stimuli) and the other half covers the domain of hyperactivity/impulsivity. Parents were asked to compare their child to other same-age children and rate their child on a 7-point Likert scale from far below average (-3) to far above average (3). Ratings were averaged. A Chinese version has been validated for Chinese children in Hong Kong (38). The alphas of SWAN in this study across three time points were respectively.93,.93, and.94.

#### Parental stress

The Parenting Stress Index Short Form (PSI-SF) was used to measure parental stress (39). A total of 36 items covers three dimensions including parental distress (PD), parent-child dysfunctional interaction (PCDI), and difficult child (DC). Parents rated the items on a 5-point Likert scale from strongly disagree (1) to strongly agree (5). Ratings were summed. A Chinese version has been validated for Chinese parents in Hong Kong (40). The alphas of PSI-SF in this study across three time points were respectively.94,.94, and.92.

#### Anxiety

The anxiety subscale of the Hospital Anxiety and Depression Scale (HADS-A) was used to measure affective and behavioral symptoms of anxiety (41). Example sentences of the 7-item scale include “I get a sort of frightened feeling as if something awful is about to happen” and “I feel restless as I have to be on the move”. Parents rated the items on a 4-point Likert scale from 0 to 3, with anchors varying between items (e.g., from “Very much indeed” to “Not at all”). A Chinese version has been validated with good psychometric properties (42). The alphas of HADS-A in this study across three time points were respectively.80,.81, and.79.

#### Depression

The 10-item version of the Center for Epidemiologic Studies Depression Scale (CES-D) was used to measure affective symptoms of depression (43). Example sentences include “I felt everything I did was an effort” and “My sleep was restless”. Parents rated the items on a 4-point Likert scale from none of the time (0) to most of the time (3). A Chinese version has been validated with good psychometric properties (44). The alpha of CES-D in this study was.81. The alphas of CES-D in this study across three time points were respectively.81,.85, and.79.

#### Family functioning

The Family Adaptation, Partnership, Growth, Affection, Resolve scale (APGAR) was used to measure the satisfaction of perceived family functioning (45). Example sentences of the 5-item scale include “I am satisfied that I can turn to my family for help when something is troubling me” and “I am satisfied with the way my family and I share time together and issues about money”. Parents rated the items on a 3-point Likert scale from hardly ever (0) to almost always (2). A Chinese version has shown high reliability and construct validity for Chinese parents and general population in Hong Kong (46). The alphas of APGAR in this study across three time points were respectively.84,.89, and.89.

#### Quality of sleep

The self-administered version of the Insomnia Severity Index (ISI) was used to assess sleeping quality (47). The 7-item scale assesses the severity of initial, middle and late insomnia, distress about sleep difficulties, interference of insomnia with daytime functioning, and notice of sleep problems by others. Parents rated the items on a 4-point Likert scale from not at all (0) to extremely (2). A Chinese version has been validated with good psychometric properties (48). The alphas of ISI in this study across three time points were respectively.84,.82, and.82.

#### Program satisfaction

Participants were invited to complete the program satisfaction questionnaire after the completion of the online program. It included four items relating to program structure, including program duration, length of video content, length of audio content, and frequency of zoom meetings on a 5-point scale. They were further invited to indicate the level of helpfulness in a 5 point scale, ranging from 0 (very unhelpful) to 4 (very helpful). Details of the questionnaire are provided in **Appendix 2**.

### Statistical analysis

One parent from the intervention group had missing data at T1 and T2. R package *mice* was used to impute the missing data. Chi-squared difference tests and *t*-tests were performed to examine demographic differences between groups. Repeated measure ANCOVAs were performed to examine differences in outcomes groups across time with parents’ educational level controlled for. R package *jmv* was used to analyze data.

For qualitative analysis, interview transcriptions were coded into themes relevant to parents’ experience in the program, especially those corresponded to the outcomes, and their suggestions of program improvement for the main study. Inter-rater reliability ensured the validity of codes and findings. Any significant inconsistencies were resolved by open discussion within the research team with coders.

## Results

### Demographics

As shown in **Table 1**, 23 parents in the intervention group and 20 parents in the control group were allocated after randomization. The majority was mothers (*n* = 40, 93.02%), married (*n* = 36, 83.72%), had ADHD children without comorbidities (*n* = 22, 51.16%). Their gender, marital status, and children comorbidity status did not differ between groups. On average, they were 42.26 (*SD* = 5.27) years old, received post-secondary education (*M* = 3.42, *SD* = 1.24), had 2.79 members in the family (*SD* = .80). However, educational level, *t*(41) = -2.154, Δ*M* = -.783, 95% CI [-1.516, -.049], *P* = .037, was significantly higher in the intervention group. It was identified as covariate in subsequent analyses.

### Program satisfaction and participation

Satisfaction was only measured in the intervention group at T1, as summarized in **Table 2**. On a 5-point Likert scale, they were satisfied with the course duration, [*M* (*SD*) = 3(.69)], length of video content[*M* (*SD*) = 3.05(.49)], and length of audio content[*M* (*SD*) = 3.09(.53)], being neither too long nor short. However, participants found the weekly zoom meetings were too frequent[M (SD) = 3.41(.73)]. Across the two groups of both the intervention and control group, the overall participation rate of four weekly Zoom meetings was 55.81%. More than half of all participants (67.44%) attended at least two meetings of weekly one-hour online group sessions.

**Table 2:** Perceived usefulness of all mindfulness practices in the program.

Practice	*M* (*SD*)
Course structure
Duration	3.00 (.69)
Length of video content	3.05 (.49)
Length of audio content	3.09 (.53)
Frequency of zoom meetings	3.41 (.73)
Satisfaction towards mindfulness exercises	
Mindful breathing	3.00 (.62)
Mindful eating	2.36 (.85)
Mindfulness of breathing and sound	2.73 (.70)
Mindful breathing together with a child	2.50 (1.10)
Body scanning	3.14 (.71)
Mindful stretching	3.27 (.70)
Child–parent stretching	2.50 (1.06)
Three-minute breathing	2.95 (.74)
Mindfulness of sound and thoughts	3.00 (.62)
Mindful on soles of the feet	2.91 (.87)
Befriending	2.95 (.90)

Given the wait-list control design, data was only collected in the intervention group. N = 23.

Although participants thought all practices were useful, there were some differences in the relative usefulness between difference practices, *F*(10, 200) = 5.725, *P* <.001. They believed that the most useful practice was mindful stretching, *M* (*SD*) = 3.27 (.70). It was perceived as more useful than mindful eating, *t*(20) = 5.396, *P* = .001, *M* (*SD*) = 2.36 (.85), mindfulness of breathing and sound, *t*(20) = 3.990, *P* = .023, *M* (*SD*) = 2.73 (.70), mindful breathing together with a child, *t*(20) = 4.250, *P* = .013, *M* (*SD*) = 2.50 (1.10), and child-parent stretching, *t*(20) = 4.250, *P* = .013, *M* (*SD*) = 2.50 (1.06). It was followed by body scan, *t*(20) = 4.202, *P* = .015, *M* (*SD*) = 3.14 (.71), and mindful breathing, *t*(20) = 4.240, *P* = .013, *M* (*SD*) = 3.00 (.62), viewed as more useful than mindful eating. Other practices were thought to be as useful as any other.

### Quantitative outcomes

**Table 3** summarized the means, standard deviations, and results from repeated measures ANCOVAs. Time effect was significant between T0 and T1, between T0 and T2, and across T0, T1, and T2 for SWAN with large effects. Between T0 and T2 and across T0, T1, and T2, the interaction between time and group had a medium to large effect on the reduction of SWAN, with larger reductions of score in intervention group than that of control group over time.

**Table 3:** Repeated measures ANCOVAs between intervention and control groups across T0, T1, and T2 after controlling for educational level.

Outcome	Timepoint	Intervention	Control	Effect	T0 vs T1			T0 vs T2			T0 vs T1 vs T2		
		*M* (*SD*)	*M* (*SD*)		*F*	*d*	*P*	*F*	*d*	*P*	*F*	*d*	*P*
APGAR	T0	4.61 (2.46)	4.50 (3.19)	Time	.103	.110	.750	.038	.063	.847	.161	.127	.851
	T1	4.65 (2.50)	4.10 (3.46)	Group	.018	.000	.893	.051	.063	.822	.076	.090	.784
	T2	5.00 (2.24)	4.40 (3.38)	Time*Group	.182	.142	.672	.307	.180	.583	.206	.142	.814
CSED	T0	11.91 (5.46)	12.80 (5.28)	Time	1.043	.320	.313	.136	.110	.715	.611	.247	.545
	T1	11.22 (5.94)	11.30 (6.48)	Group	.004	.000	.953	.017	.000	.897	.025	.063	.876
	T2	9.70 (4.70)	10.30 (4.65)	Time*Group	.078	.090	.782	.169	.127	.683	.085	.090	.919
HADS	T0	9.13 (3.96)	9.30 (4.27)	Time	1.264	.358	.268	.350	.191	.557	.616	.247	.542
	T1	7.87 (3.82)	7.90 (3.37)	Group	.175	.127	.678	.398	.201	.532	.345	.191	.560
	T2	7.74 (4.09)	7.65 (2.85)	Time*Group	.000	.000	.988	.052	.063	.820	.048	.063	.953
PSI	T0	118.78(25.21)	123.45(19.63)	Time	.218	.142	.643	.031	.063	.861	.146	.127	.864
	T1	116.57(20.21)	121.85(25.04)	Group	1.903	.434	.175	1.357	.369	.251	1.692	.414	.201
	T2	115.70(20.63)	118.75(19.07)	Time*Group	.034	.063	.854	.132	.110	.718	.179	.127	.837
SWAN	T0	1.32 (.75)	1.19 1.03)	Time	7.700	.876	.008	8.727	.934	.005	5.390	.735	.006
	T1	.97 (.92)	1.06 (.80)	Group	.008	.000	.928	.223	.155	.640	.431	.211	.515
	T2	.87 (.96)	1.18 (.71)	Time*Group	2.583	.510	.116	7.735	.879	.008	3.356	.578	.040
SWAN (Inattention)	T0	1.45 (.79)	1.27 (.99)	Time	5.918	.770	.020	6.909	.830	.012	3.970	.629	.023
	T1	1.09 (.98)	1.28 (.84)	Group	.174	.127	.679	.483	.220	.490	1.210	.346	.278
	T2	1.00 (1.00)	1.40 (.69)	Time*Group	4.798	.692	.034	9.289	.962	.004	4.298	.655	.017
SWAN (Hyperactivity)	T0	1.19 (.87)	1.11 (1.15)	Time	8.087	.899	.007	8.372	.915	.006	5.724	.756	.005
	T1	.90 (.95)	.84 (.86)	Group	.045	.063	.834	.054	.063	.818	.047	.063	.829
	T2	.73 (1.04)	.96 (.87)	Time*Group	.616	.247	.437	4.303	.655	.045	1.888	.434	.158
ISI	T0	11.09 (5.43)	11.45 (5.98)	Time	.126	.110	.724	.871	.293	.356	.967	.314	.385
	T1	9.57 (4.52)	10.70 (4.94)	Group	.025	.063	.876	.001	.000	.981	.006	.000	.939
	T2	10.43 (5.16)	10.30 (4.70)	Time*Group	.049	.063	.825	.020	.000	.889	.070	.090	.932

Results of the subscales revealed that the effect on SWAN was largely driven by the program effectiveness on the perceived inattentive behaviors specifically. The moderate to large effect allowed parents in intervention group to see their children’s inattentive behaviors closer to those among average children while those in control group perceived the behaviors further away from the norm. The medium effect on hyperactivity was significant only between T0 and T2, but the trend was obvious considering the small sample size.

There were positive changes in depression, anxiety, parenting stress, family functioning, and quality of sleep in the participants from intervention group from T0 to T1, and T1 to T2. However, similar changes were also reported in waitlist control group. There were no significant time and group interaction effects in all other outcome measures.

### Qualitative findings

Nine parents participated in the online interviews, and their experiences are categorized into three main areas: help-seeking intention, personal changes and suggestions for improvement.

#### Help-seeking intention

Some parents reported that they joined the program because they expected the benefits of mindfulness to improve their emotional management. Perceived benefits before joining the course included managing fatigue (Parent 2, 4), managing worries (Parent 3), stabilizing emotions (Parent 6) and relaxing (Parent 7). Some participants explicitly mentioned their wishes to gain insights into parenting (Parent 4, 5, 9). Besides, others identified specific reasons for enrolling this program, namely shorter duration with high flexibility (Parent 1) and free of charge (Parent 4, 7).

Parents revealed their difficulties in managing emotions (Parent 1, 2, 3), and all parents reported anxiety and parenting stress to different extent, and some reported having physiological conditions arising from stress.

*I am so nervous, and at that time I am very worried and I have to plan everything in my mind constantly…. and I scold him (son) a lot. I ask myself why I would be that emotional and scold at him,… Sometimes I would be very emotional,…and I would close the door and scream. I feel so overwhelmed, like why things just loop around.* (Parent 3)

*There is so much parenting pressure. And I think I am a bit lost, and I want to find something, or maybe take some mindfulness course to help myself, at least to ease my state of tension first.* (Parent 4)

#### Personal changes

Parents noticed changes such as positive management of their anger and anxiety. Some parents further explicitly mentioned that they had developed a habit of being mindful of their bodies and emotional states.

*When I am relieved and I think that I can spread my love to others, my feelings changed, and I am not so angry. Actually I think my child has said much the fact that he was angry about himself, … and why he was absent-minded. … After noticing this, my feelings are much better.* (Parent 1).

*when I moved them to the soles of the feet, I stopped coughed, and my throat had nothing wrong. This is one of the few things I found amazing after this course … when I was so focused on doing mindfulness practice, even my physical illness and the symptoms have some improvement.* (Parent 1)

Some parents said they experienced more time of calmness after the program. Some parents also reported that they were reminded to take care of themselves before having the capacity to take care of their children.

*If I don’t take care of myself, how can I take care of my child? If I leave myself to be unhappy, just letting my muscles sore, … I would not have the endurance to do it. So, this is a reminder.* (Parent 9)

*If my child was not in a good condition, or did minor something, it would trigger my mood easily. I would get irritated. And now I have learned, whenever I have 10-minute travel, I would close my eyes and breath, feel my body and sense the fatigue in my shoulder. …I can connect with myself again, not only living for my family and children* (Parent 7).

In addition to the personal changes of the parents, some further expressed they noticed changes in their parent-child relationships, and the style of communication. Some parents also said that their family members were less tense after the program. At the same time, parents noticed that their children were more responsive, emotionally stable, and initiative, and their children talked more about their thoughts and emotions.

*I started something, like “What is your preference? What do you want?” … it surprises me that actually he didn’t know what he wants. … So, we talked more. Rather than giving orders at that moment, and (expected that) he obeys.* (Parent 4)

*Now I will try my best to give him more chances to express himself. … (On the other hand), he would listen to me, and he gives feedback like, “oh, I know mom is busy”, or “I know mom is tired.” He starts to learn to care about his mom, and say things that, are caring.* (Parent 2)

#### Suggestion for improvement

Some parents made suggestions to the improvements on videos, mindfulness practice and audio files, and weekly zoom sessions with instructors. For the video, most parents reported that the video lengths are suitable for busy parents. Parents also said that the contents were stimulating and gained insight from the speakers’ sharing. However, the videos can be improved by including more animation to attract children to view, incorporating parenting components, and include more content to teach their children with ADHD to do mindfulness activities. Most parents reported that the instructions were easy to follow for the audio recordings and mindfulness practice, and the length was optimal. The tasks were manageable and achievable and were broken down into smaller steps. The minimal rules instructed in the mindfulness exercises allow the parents to have the flexibility to adjust according to their individual needs, and the inclusion of various exercises and options also provides flexibility for the parents. As the audio files are sent to the parents, they can listen to them whenever they want.

Two parents found that the 1-hour session was too long for parents to commit to if it was not the children’s summer holidays and that the meeting time does not suit working parents’ schedules well. A parent emphasized that some participants in the zoom meetings remained silent and disengaged, although the instructor has made much effort to encourage them to turn on the camera and share their parenting difficulties. For example, “(E)*ven some parents joined (the online meetings) every time, they still remained silent. It is difficult to build up trust and share more personal experiences* (Parent 4). “*Some parents may not have a suitable and quiet environment to express themselves … If there are not many parents ready to share, the time may be too much*.” (Parent 2) Instructors should pay more attention to engage participants in inquiry and exchange. Further improvements, such as more detailed descriptions of the mindful movements, can help parents understand how they should follow the mindful stretching. The instructions were too fast for some parents that they experienced difficulties catching up. One parent has suggested that the inclusion of shorter practices could be designed in future programs, as it was challenging to practice long activities in their workplace.

## Discussion

This study used a mixed methods to investigate the effects of an online mindfulness-based program. We investigate whether an online program is acceptable to the parents of children with ADHD and test if can produce similar benefits to regular face-to-face mindfulness-based programs. The use of online format can largely improve the accessibility of mental health care, as many parents may not be able to join a face-to-face mindfulness-based program due to their busy life schedule, and relatively expensive costs and wait lists of face-to-face treatments. In this pilot study, a waitlist randomized controlled trial was used and the outcomes provided preliminary evidence on the positive outcomes on both parents and their children with ADHD.

The results provided some evidence for supporting the hypotheses of this study. We found a significant reduction of ADHD symptomology after their parents completed the program, as hypothesis 2 has been supported. Previous studies of MBPs on children with ADHD and their parents were face-to-face programs (22, 23), and this study targeted parents of children with ADHD and applied an online format. In clinical practice, it would be challenging to engage the children with ADHD in an online program. However, this study outcome suggested that children with ADHD could benefit after their parents have learned and practice mindfulness first by themselves, and later engaging their children to do the exercises together at home. The effect size in ADHD symptomology is similar to a previous study of a family-based mindfulness program by the research team (23). Although the data did not support hypothesis 1 in this study, parents in intervention group reported a reduction parenting stress. The lack of time x group effect may be attributed to the small sample size or a short intervention length of four weekly treatments.

The positive experience in parents received support from the qualitative interviews, as many parents shared their improvements in emotion regulation. Parents expressed that their help seeking intentions were enhanced by the online mindfulness-based program, and they perceived the program as helpful in not only improving their mental health, but also in modifying the dysfunctional interaction patterns that happened in many families of children with ADHD. In the qualitative interviews, some parents explicitly shared their benefits, namely an enhanced awareness to their bodies and difficult emotions such as anger and anxiety in managing the challenging behaviors of children with ADHD. Regarding the quantitative results, the effect sizes of the intervention group from T0 to T2 was d = 0.13, while those of the waitlist control group was d = 0.24. The result of the waitlist control group was similar to the results of a pervious face-to-face family MBP (23). It would be advisable to conduct further studies with an adequate sample size to examine whether an online MBP can yield a similar effect to face-to-face program with comparable intensity.

As mentioned, online mindfulness-based intervention has received growing attention but its application in clinical populations remained limited. This study is built on the successful experience of an online mindfulness-based program for healthy parents, which was a purely self-help program (34). In this study, we integrated low intensity professional support (consisting of four weekly, hourly sessions) with purely self-help online modules, resulting in significant positive outcomes. When comparing it to the typical structure of most MBPs, which consists of weekly meetings lasting 2 hours or longer, with only one session only per week, the adapted program structure that combines brief daily online psychoeducation videos with audio mindfulness guidance may be more suitable to many parents of children with ADHD. This is because these parents often have busy daily schedules and some of them also face limitations in their short attention span.

Overall, the participants showed satisfaction about the program structure with most related scores being above 3 points, including the duration of course, the length of video content, and the length of audio content. Many participants appreciated the support they received from experienced MBP instructors. Only two parents in our qualitative data expressed their reluctance to spend four hours in total to the online group sessions, and in the satisfaction survey, the frequency of online meetings also have relatively lower score than other items. Further studies should adjust the number or frequency of sessions of online meetings and explore the effects of different options of professional support, including formats and dosage, and their mapping of the needs of different clinical populations.

This study is unique in integrating parent-only mindfulness exercises with parent-child mindfulness exercises. Based on the satisfaction scores of different mindfulness exercises, the research team decided to further expand the choice of videos and audio files. When comparing the levels of satisfaction with parent-only exercises and parent-child exercises, the results indicated that parents derived greater benefits from parent-only exercises. This suggests that engaging children to participate in mindfulness exercises together with their parents may pose challenges. In response to the feedback from qualitative interviews that some parents requested to include child only exercises, the research team decided to add additional videos and audio files specifically designed for children as an alternative approach. This way, children with ADHD can choose to practice mindfulness either on their own or with their parents. The aim is to ensure families with different needs can still benefit from the various modules offered in the online program.

In addition, this program involved children with ADHD to do mindfulness-related exercises together with their parents, which could also provide novel insights of developing such interventions. Apart from the reduction of ADHD symptomology among ADHD children of participants according to the quantitative data, children with ADHD only engaged in a later part of the whole program, but parents in the qualitative interviews reported that their children were more responsive, emotionally stable, and initiative to communicate more about their thoughts and emotions, which might be improved through the process that they and their children were together to take efforts. Studies should further investigate the length of parents actually implementing mindfulness exercises with their children, which can give inspirations of the impacts of MBP intervention on children’s ADHD symptoms. Besides, further studies might also adjust the program to contain more content to attract children’s attention and make it easier to follow the instructions to do mindfulness-based exercises.

Further studies should explore the mechanisms of change in MBPs for parents. Recent studies have explored the role of expressed emotions (EE) among parents of children with ADHD children (24, 49). EE was originally developed to understand family environments characterized by criticism, hostility and over-involvement, and its role in predicting clinical outcomes for individuals with serious mental illness, particularly adults with schizophrenia and their relapse (50). High EE refers to frequent, sustained, and uncontrollable family stress that may interact with individual biological vulnerabilities, contributing to promoting the recurrence of illness (51). In this pilot study, the MBP demonstrated some reduction in parenting stress and children’s ADHD symptoms. These results could be explained by a previous study where young adults with psychosis reported significant reductions in their parent’s EE after their parents completed a MBP (52). This reduction in EE may be a potential mechanism behind the positive changes in MBP for parents. One potential benefit of mindfulness exercises is their ability to reduce negative emotional responses and promote effective emotion regulation (53, 54). Additionally, parenting intervention has been shown to significantly reduce EE among parents of children with ADHD (55). It suggests that MBPs may improve the mental health outcomes of children with ADHD and positively impact the family environment. However, the use of EE in child psychopathology, particularly in Asian families is limited, apart from a protocol for a study on ADHD in Japan (56). This pilot study did not examine the mechanisms of EE in Chinese families with ADHD children, and further studies on EE in the context of ADHD among Chinese families are warranted.

In spite of the encouraging result of the study, the research team also identify some limitations of this study. First, the small sample size largely limits the level of evidence of this study and interpretation of the finding should be cautious. Other selected outcomes of the study including parenting stress and mental health, family functioning did not reach the level of statistical significance, reflecting the limitations of sample size. With a power of.80 and alpha at.05, the same three-timepoint randomized controlled trial design requires a sample size of 146 or more to reach the statistical significance. The research team has submitted a funding proposal successfully and a large-scale study comparing the outcomes between an ordinary psychoeducation with mindfulness-based program in this target group has kicked start in early 2024. Second, the waitlist control design limited the differentiation of the effectiveness between mindfulness-related contents and general psychoeducation. In our large-scale clinical trial, active control group is included. Further studies may consider the use of a two-arm or three-arm trial with an active control group.

Third, due to the nature and limited resources of a pilot study, this study selected parent self-reported measures for outcome study. It is unclear whether the change in child ADHD symptomology is due to the perception of parents after their completion of mindfulness-based program or the change in child behaviors. Further study should use multiple measures, and the uses of behavior observation, psychological tests and biomarkers are possible options. As mentioned, some studies applied Five Minute Speech Sample for measuring parent’s expressed emotions (49), attention network test and self-regulation tests for children (23, 57) that have been used in parenting intervention or mindfulness-based programs should be considered. Such situations are likely to explain the similar positive results of the waitlist group with mindfulness-based intervention. Further study may consider conducting studies in other situations to differentiate potentially specific effects of mindfulness-related treatments. Besides, this pilot study chose to recruit healthy parents based on their self-report instead of evaluating the ADHD symptoms of them. Given the genetic inheritance patterns of ADHD (57), further studies should understand how the parents’ ADHD symptoms are diagnosed. Thus, when participating in the MBP intervention, the improvements of their ADHD symptoms might result in a more accepting parental demeanor and be helpful to explore the mechanisms of changes in the MBP intervention.

## Conclusions

In this mixed methods study, preliminary evidence is identified in applying online mindfulness-based program in treating parents and children with ADHD. There is a pressing need to investigate the effective use of technology in promoting mental health care to people with clinical conditions. Online programs are possible options for parents and family caregivers who have been burdened by providing care to their family members in need and find themselves incapable of spending time and effort to participate in a face-to-face program. Further study should explore the integration of self-help intervention with low intensity professional support and needy children, individuals and families can get adequate care in facing mental health challenges.

## Data availability statement

The raw data supporting the conclusions of this article will be made available by the authors, without undue reservation.

## Ethics statement

The studies involving humans were approved by PolyU Institutional Review Board. The studies were conducted in accordance with the local legislation and institutional requirements. The participants provided their written informed consent to participate in this study.

## Author contributions

HL: Conceptualization, Funding acquisition, Investigation, Methodology, Project administration, Writing – original draft, Writing – review & editing. JL: Data curation, Formal analysis, Investigation, Writing – original draft. ZZ: Conceptualization, Formal analysis, Investigation, Writing – original draft, Writing – review & editing. MC: Data curation, Investigation, Writing – review & editing. SC: Conceptualization, Investigation, Writing – review & editing. EW: Conceptualization, Investigation, Writing – review & editing. SB: Conceptualization, Investigation, Writing – review & editing. EC: Conceptualization, Funding acquisition, Writing – review & editing.
