# Supplementary material for: Effects of an online mindfulness-based program for parents of children with attention deficit/hyperactivity disorder: a pilot, mixed methods study
Source: Front Psychiatry. 2024 Apr 24;15:1376867. doi: 10.3389/fpsyt.2024.1376867 (PMC11131379; doi:10.3389/fpsyt.2024.1376867)
Supplement: Supplementary file 1 [file DataSheet_1.docx]

Appendix 1 Online mindfulness-based program for parents of children with ADHD

| **Module** | **Session** | **Formal Practice** | **Informal Practice** | **Video content** |
| --- | --- | --- | --- | --- |
| 1. **mindfulness and attention** | 1 | Mindfulness & breathing (I) | Observing a daily routine | Managing distraction |
|  | 2 | Mindfulness & breathing (II) | Observing a daily routine | Mindful parenting (1) |
|  | 3 | Mindful eating | eating a meal or snack | Parenting with a beginner’s mind (2) |
|  | 4 | Mindfulness of breathing & sound (I) | Observing child with a beginner’s mind | The intention of mindfulness of sounds |
|  | 5 | Mindfulness of breathing & sound (II) | Mindful breathing together with a child | Mindful breathing with a child |
|  | 6 | **+Mindfulness & breathing (Child)** | **Mindful breathing together with a child** |  |
|  | 7 | self-selected | self-selected | / |
| **Week 1 Online meeting**  Orientation, Expectations of the program, Live Mindfulness Exercise lead by instructor, Inquiry, Q & A | | | | |
| 1. **mindfulness and**   **physical sensation** | 8 | Body scan (I) | Observing a child with beginner’s mind | Mindfulness of physical sensation in parenting |
|  | 9 | Body scan (II) | Observing physical sensation in child-parent interaction | Mindfulness of physical sensation in child-parent interaction |
|  | 10 | Mindful stretching (I) | Observing physical sensation in child-parent interaction | Being with unpleasant physical sensations |
|  | 11 | Mindful stretching (II) | Child-parent stretching | Accepting limits in mindful stretching |
|  | 12 | Mindful stretching (III) | Child-parent stretching | Accepting limits of our children with special needs |
|  | 13 | **+ Body scan (Child)** | **Sharing the awareness of physical awareness** |  |
|  | 14 | Self-selected | self-selected | / |
| **Week 2 Online meeting**  Live Mindfulness Exercise led by instructor, Inquiry, Discussion of guiding child mindfulness exercise at home | | | | |
| 1. **mindfulness and**   **parental stress** | 15 | Breathing space | 3 min breathing | The intention of developing breathing space |
|  | 16 | Breathing space | 3 min breathing | Developing space in a parenting |
|  | 17 | Mindfulness of sounds & thoughts (I) | 3 min breathing | The intention of mindfulness of thoughts |
|  | 18 | Mindfulness of sounds & thoughts (II) | 3 min breathing | Thoughts in mindful parenting |
|  | 19 | Mindfulness of sounds & thoughts (III) | Guiding a breathing for your child | Awareness of speaking and listening and child-parent interaction |
|  | 20 | **+ Distraction in child-parent exercise** | **Sharing the awareness of physical awareness** |  |
|  | 21 | Self-selected | self-selected | / |
| **Week 3 Online meeting**  Live Child-Parent parallel Mindfulness Exercise led by instructor, Inquiry, Half way review | | | | |
| 1. **mindfulness and self-care** | 22 | Soles of the feet (I) | 3 min breathing | Strong emotions in parenting |
|  | 23 | Soles of the feet (II) | 3 min breathing | Accepting our limitations in parenting |
|  | 24 | Befriending (I) | 3 min breathing | Sending kind wishes to ourselves |
|  | 25 | Befriending (II) | Mindful games in child parent interaction | Sharing mindfulness with your child |
|  | 26 | Review and reflection | self-selected | Everyday reminders of mindful parenting |
|  | 27 | **+ Befriending exercise (Child)** | **self-selected** |  |
|  | 28 | self-selected | self-selected | / |
| **Week 4 Online meeting**  Live Mindfulness Exercise led by instructor, Inquiry, Discussion on application of mindfulness in future life | | | | |

Appendix 2 Program Satisfaction Questionnaire

Please indicate your level of satisfaction toward the online program that you have just completed using a score of 1 to 5.

About the course structure

1. Duration (28 days) 1 (too short) 2 (a bit too short) 3 (just suitable) 4 (a bit too long) 5 (too long)
2. Length of video content (5 to 10 minutes each day) 1 (too short) 2 (a bit too short) 3 (just suitable) 4 (a bit too long) 5 (too long)
3. Length of audio content (5 to 10 minutes each day) 1 (too short) 2 (a bit too short) 3 (just suitable) 4 (a bit too long) 5 (too long)
4. Frequency of zoom meetings (four times, once per week) 1 (should be more frequent) 2 (should be a bit more frequent) 3 (just suitable) 4 (a bit too frequent) 5 (too frequent)

About the satisfaction towards mindfulness exercises

1. Mindful breathing 0 (very unhelpful) 1 (a bit unhelpful) 2 (average) 3 (quite helpful) 4 (very helpful)
2. Mindful eating 0 (very unhelpful) 1 (a bit unhelpful) 2 (average) 3 (quite helpful) 4 (very helpful)
3. Mindfulness of breathing and sound 0 (very unhelpful) 1 (a bit unhelpful) 2 (average) 3 (quite helpful) 4 (very helpful)
4. Mindful breathing together with a child 0 (very unhelpful) 1 (a bit unhelpful) 2 (average) 3 (quite helpful) 4 (very helpful)
5. Body scanning 0 (very unhelpful) 1 (a bit unhelpful) 2 (average) 3 (quite helpful) 4 (very helpful)
6. Mindful stretching 0 (very unhelpful) 1 (a bit unhelpful) 2 (average) 3 (quite helpful) 4 (very helpful)
7. Child–parent stretching 0 (very unhelpful) 1 (a bit unhelpful) 2 (average) 3 (quite helpful) 4 (very helpful)
8. Three-minute breathing 0 (very unhelpful) 1 (a bit unhelpful) 2 (average) 3 (quite helpful) 4 (very helpful)
9. Mindfulness of sound and thoughts 0 (very unhelpful) 1 (a bit unhelpful) 2 (average) 3 (quite helpful) 4 (very helpful)
10. Mindful on soles of the feet 0 (very unhelpful) 1 (a bit unhelpful) 2 (average) 3 (quite helpful) 4 (very helpful)
11. Befriending 0 (very unhelpful) 1 (a bit unhelpful) 2 (average) 3 (quite helpful) 4 (very helpful)

Appendix 3 Qualitative interview guide

Semi-structured interviews were conducted based on the following questions to explore the parents’ experiences related to their participation in the program:

1. General questions about help seeking.

a. What makes you join the online mindfulness program? How would you describe yourself before the group?

b. What kind of services have you been looking for managing your child with ADHD and your parenting stress? (including self-help, individual counseling, group counseling)

2. Personal changes.

a. What changes, if any, have you been noticed in yourself after the online mindfulness program? (For example, are you doing, feeling, or thinking differently from the way you did before? What specific things, if any, have you got from the program?)

b. What changes, if any, have you been noticed in your children and your family after your participation in the online mindfulness program? (For example, are they doing, feeling, or thinking differently from the way they did before?)

c. Has anything changed for the worse for you and for your family since you joined the group?

d. Is there anything that you wanted to change but hasn’t since you joined the group?

3. Suggestions for improving the program.

Please share, if any, about the areas we can improve for coming participants of the program so that they can benefit most:

a. About the video (psychoeducation and intentions of the practice)

b. Mindfulness practice and practice audio files

c. Weekly zoom sessions with mindfulness-based instructor

d. Other arrangements of the course (recruitment/promotion, follow-up of research assistants, etc.)

4. Any thoughts about the program that you may want to share with the research team.
